# Navigating the Intersection of Radiofrequency Microneedling and Surgical Facelifts: Scoping Review

**DOI:** 10.2196/78385

**Published:** 2026-04-27

**Authors:** Mia Panlilio, Rebecca Bolen, Olnita Martini, Alexa Bonk, John Tedesco

**Affiliations:** 1 Department of Biomedical Sciences, College of Osteopathic Medicine, Rocky Vista University, 8401 S Chambers Rd, Englewood, CO, 80112, United States, 1 9253236431; 2 College of Osteopathic Medicine, Oklahoma State University, Tulsa, OK, United States

**Keywords:** radiofrequency, microneedling, facial, laxity, facelift

## Abstract

**Background:**

Optimal management of facial skin laxity requires a nuanced approach by health care providers working in aesthetics. Radiofrequency microneedling (RFMN) devices have emerged as a popular noninvasive treatment for facial rejuvenation and improving skin laxity. While RFMN has demonstrated efficacy in enhancing skin tightening and complementing aesthetic procedures, its long-term impact on subsequent surgical facelifts remains uncertain.

**Objective:**

The objective of this scoping review is to explore the interplay between RFMN and surgical facelift outcomes, with a focus on potential complications such as excessive skin tightening, dermal scarring, and altered tissue planes that may pose surgical challenges.

**Methods:**

A search using PubMed and Google Scholar was conducted, and articles were selected from peer-reviewed journals based on specific inclusion and exclusion criteria. Only articles available in English were selected. In total, 21 articles were included in this scoping review.

**Results:**

Papers included in this review discussed the mechanisms of action involved with RFMN, RFMN-related tissue changes, and how these changes could impact future facelift procedures. Most of the papers found that RFMN may drastically alter multiple tissue planes involved in facelift procedures due to collagen deposition through multiple tissue layers and increased tissue fibrosis. Patient factors influencing the effectiveness of RFMN and its role in facial rejuvenation were also examined, emphasizing the importance of navigating patient-specific demographics as a future consideration when creating an individualized treatment plan for each patient.

**Conclusions:**

Patients should be informed that RFMN may lead to dermal fibrosis, tissue adhesions, and altered superficial musculoaponeurotic system composition, which could interfere with future facelift procedures and the patient’s desired treatment goals. This emphasizes the importance of detailed discussion between the patient and health care provider to improve pretreatment consultation, increase patient education, and set realistic expectations. Further research is needed to determine optimal timing and treatment strategies for patients considering both RFMN and surgical facelifts to achieve the best aesthetic outcomes.

## Introduction

Radiofrequency (RFMN) microneedling is a minimally invasive procedure that combines mechanical injury and thermal stimulation via tiny microneedles with radiofrequency (RF) energy to induce collagen remodeling [1]. The microneedling component creates controlled microtraumas, triggering a postinflammatory cascade that promotes neocollagenesis, elastin production, and angiogenesis. RF energy delivered through the microneedles then generates thermal coagulation within the dermis and hypodermis to induce collagen denaturation and subsequent contraction of tissue for skin tightening benefits [2]. This fractional approach allows for targeted treatment while preserving surrounding tissue, reducing recovery time [3]. Compared to traditional microneedling, evidence suggests that RFMN brings about greater improvements in aged skin, likely by eliminating senescent fibroblasts and increasing the number of nonsenescent fibroblasts [4].

A systematic review done in 2021 by Tan et al [5] analyzed 42 studies evaluating RFMN use across various conditions, with the largest evidence base for skin rejuvenation, followed by acne scars, acne vulgaris, striae, and axillary hyperhidrosis. A smaller number of studies were available supporting RFMN use for melasma, rosacea, cellulite, and androgenetic alopecia.

Based on the large and growing body of evidence for skin rejuvenation, RFMN devices have gained immense popularity for addressing skin laxity in patients seeking noninvasive alternatives to surgical facelifts. These devices offer treatment options for individuals outside the average age range for a facelift, those who have previously undergone a facelift, or patients desiring minimally invasive interventions [5]. Advances in RFMN technology, such as interchangeable tips with various microneedle pin configurations and dual treatment modes, allow for targeted treatments in delicate anatomical areas like the periorbital region [5]. While these technological refinements enhance customization, the process may impact deeper dermal structures critical to surgical outcomes, setting the stage for potential interference with future facelift procedures.

Long-term effects of RFMN before surgical facelifts remain unclear, raising concerns about potential complications. RFMN treatment prior to an elective facelift may have the potential to interfere with optimal facelift results due to excessive skin tightening, scarring, and damage to the dermis. This emphasizes the importance of pretreatment discussion about expectations and the adverse effects of RFMN if it is being used with a patient who is considering a facelift in the future. Given the interplay between RFMN and surgical facelifts, what are the long-term implications of RFMN for patients who may eventually pursue surgical facelift procedures? Could the very technology we’re using to delay surgery unintentionally complicate it later? This scoping review explores the anatomical and clinical intersections between RFMN and surgical facelifts for patients realistically considering either a facelift or RFMN. It draws on current literature, evolving device technology, and real-world considerations to guide thoughtful treatment planning for optimal patient outcomes.

## Methods

Searches done on PubMed and Google Scholar using the terms “skin laxity and radiofrequency microneedling,” “skin laxity and microneedling,” “skin laxity and facelift,” “facelift and radiofrequency microneedling,” and “facelift and microneedling” were conducted on January 4, 2024, and again on June 20, 2025, to account for any newly published or updated literature since the original search. The second search did not yield any new articles. Articles from peer-reviewed journals were included if they provided information on the mechanism of action of RFMN, described the techniques involved when performing a facelift procedure, or examined the effects of RFMN and/or facelift procedures on the skin. Only articles available in English were selected, and articles were excluded if they provided information on the use of RFMN on parts of the body other than the face and neck, or if they described RFMN treatment or surgical intervention unrelated to improving facial skin laxity. A summary of the inclusion and exclusion criteria are highlighted in Textbox 1. The review was conducted based on the 2005 methodology of Arksey and O’Malley [6]. The Preferred Reporting Items for Systematic Reviews and Meta-Analyses extension for Scoping Reviews (PRISMA-ScR) reporting guidelines were followed, and the completed PRISMA-ScR checklist for this review can be found as Checklist 1.

Textbox 1. **Inclusion and exclusion criteria.**

**Inclusion criteria**

- From peer-reviewed journals
- Content of article: mechanism of action of radiofrequency microneedling (RFMN); facelift procedures; effects of RFMN and/or facelift procedures on the skin
- Available in English

**Exclusion criteria**

- Content of article: RFMN being used on parts of the body other than the face and neck; RFMN treatment or surgical intervention unrelated to improving facial skin laxity
- Not available in English or an English translation

## Results

### Article Selection

**Figure 1.**
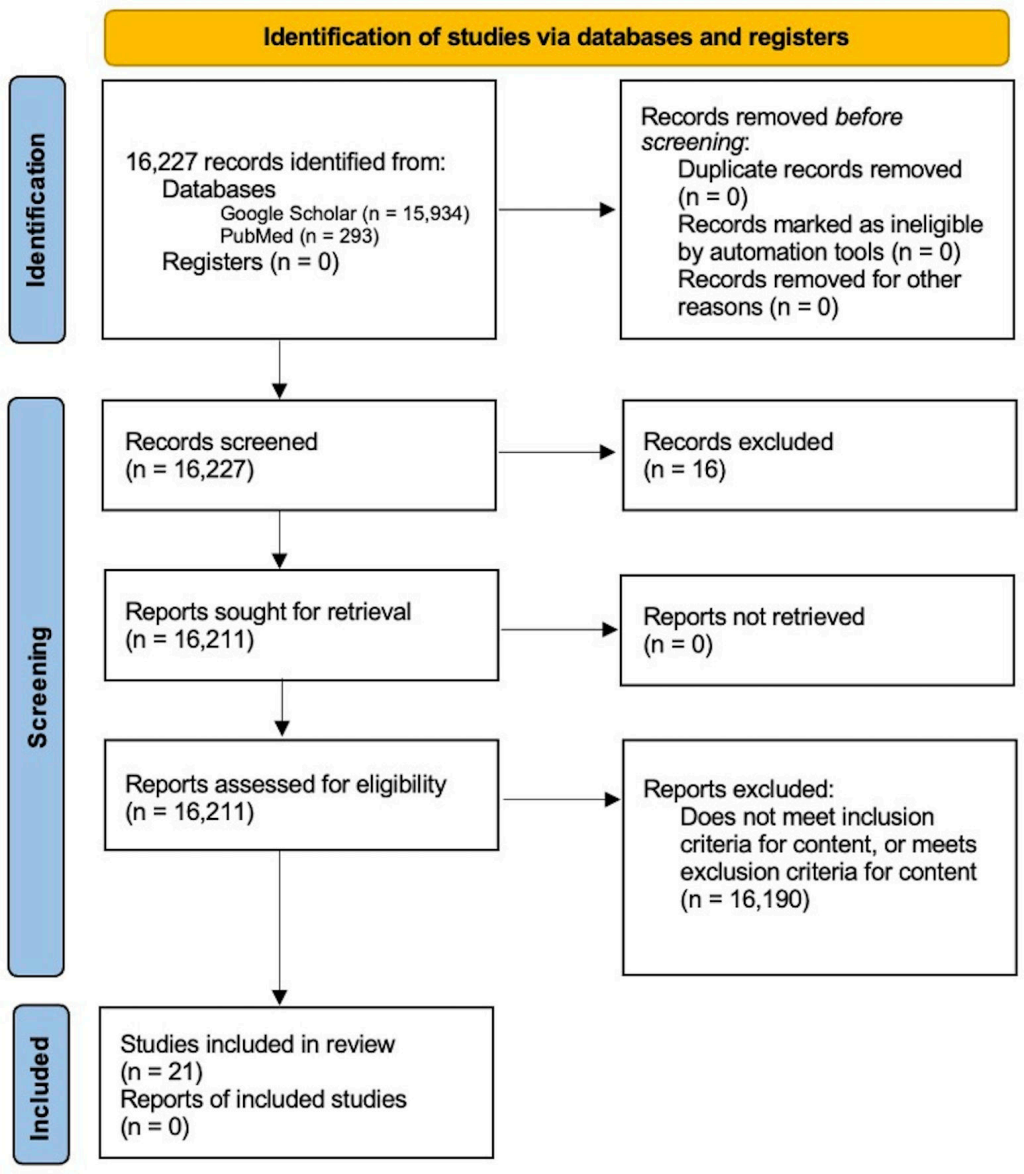
Preferred Reporting Items for Systematic Reviews and Meta-Analyses (PRISMA) diagram of article selection.

From the initial search using the selected search terms, 15,934 and 293 articles were respectively identified from Google Scholar and PubMed, for a total of 16,277 articles (Figure 1). Out of the 16,277 articles screened, 16 PubMed articles were excluded because they were not available in English. Google Scholar does not have a language screening filter, so all 15,934 articles from the initial search were still considered. After all authors screened the remaining articles for content based on the inclusion and exclusion criteria, 21 articles in total were selected for this review. A PRISMA diagram of the article selection is available below in Figure 1.

### Summary of Included Articles

After article screening was completed and the 21 articles were selected, 12 articles were identified as original research, 5 as narrative literature reviews, 2 as systematic reviews, and 1 as an educational reference article. One article, by Arksey and O’Malley [6], was used as a framework to guide writing of this review, and did not necessarily fit the inclusion and exclusion criteria for articles specifically related to the topic of this scoping review. Table 1 displays a summary of the articles mentioned, as well as their article types and study designs.

**Table 1.** Summary of included articles.

Authors (year)	Journal	Article type	Study design
Devgan et al (2019) [1]	*Otolaryngology Clinics of North America*	Literature review	Narrative review
Spataro et al (2022) [2]	*Facial Plastic Surgery Clinics of North America*	Literature review	Narrative review
Hendricks and Farhang (2022) [3]	*Journal of Cosmetic Dermatology*	Literature review	Narrative review
Hwang et al (2025) [4]	*Scientific Reports*	Original research	Split-face comparative clinical trial
Tan et al (2021) [5]	*Dermatologic Surgery*	Literature review	Narrative review
Arksey and O’Malley (2005) [6]	*International Journal of Social Research Methodology*	Original research	Not applicable
Dayan et al (2020) [7]	*Plastic and Reconstructive Surgery—Global Open*	Original research	Prospective case series (single-arm clinical study)
Arnaoutakis et al (2022) [8]	*Facial Plastic Surgery & Aesthetic Medicine*	Literature review	Narrative review
Ramaut et al (2018) [9]	*Journal of Plastic, Reconstructive & Aesthetic Surgery*	Systematic review	Systematic review
Huang et al (2014) [10]	*Biochemistry*	Basic science research	In vitro experimental study
Nguyen et al (2025) [11]	*Lasers in Medical Science*	Original article	Clinical and histologic study (prospective cohort)
Xu et al (2025) [12]	*Lasers in Surgery and Medicine*	Original research	Animal study (porcine model)
Zheng et al (2014) [13]	*Dermatologic Surgery*	Original research	Experimental histologic study
Wang et al (2025) [14]	*Lasers in Medical Science*	Original research	Pilot clinical study
Wang et al (2024) [15]	*Lasers in Surgery and Medicine*	Original research	Animal study (porcine model)
Cho et al (2024) [16]	*Skin Research & Technology*	Original research	Animal study (minipig model)
Hohman et al (2023) [17]	*StatPearls*	Reference article	Narrative review (educational)
Ghassemi et al (2003) [18]	*Aesthetic Plastic Surgery*	Original research	Anatomical cadaveric study
Demesh et al (2021) [19]	*Journal of Cosmetic Dermatology*	Original research	Clinical case series
Seo et al (2012) [20]	*Lasers in Surgery and Medicine*	Original research	Clinical and histologic study (prospective cohort)
Austin et al (2022) [21]	*Lasers in Surgery and Medicine*	Systematic review	Systematic review

### RFMN-Related Tissue Changes

While RFMN effectively improves skin laxity and wrinkle reduction, its impact on future facelift procedures remains uncertain. An article published in 2022 found that a single session of noninvasive fractional bipolar RFMN achieved approximately 37% of the skin laxity improvement seen with a surgical facelift, suggesting multiple treatments may be required for significant results [8]. However, a 2018 systematic review from the *Journal of Plastic, Reconstructive & Aesthetic Surgery* states that repeated sessions risked dermal fibrosis, particularly in the papillary dermis, potentially complicating future surgical interventions [9].

As RFMN devices introduce repeated and organized microtraumas into the skin, the depth of penetration directly impacts targeted tissue layers. Microneedling from such devices introduces a targeted mechanism of repair that excludes highly inflammatory cellular cascades, such as transforming growth factor β-1 (TGF-β1) and transforming growth factor β-2 (TGF-β2), and instead is driven through a less-inflammatory cascade via transforming growth factor β-3 (TGF-β3), a protein known to lead to fibroblast migration and collagen matrix remodeling [10]. Platelet-derived growth factor (PDGF), fibroblast growth factor (FGF), and epidermal growth factor (EGF) are all released locally in response to the microtrauma, allowing natural skin tightening via angiogenesis and collagen deposition [2]. Repeated treatments thus increase collagen deposition, which increases the risk for the development of dermal fibrosis.

A better understanding of the timeline of collagenesis following RFMN is critical when considering the interplay with surgical facelifts. Acute inflammation and early collagen deposition dominate the first week after treatment, followed by organized collagen remodeling and maturation over the ensuing 1 to 3 months [2,10]. Persistent changes in dermal structure, including fibrosis or altered tensile strength, may interfere with surgical flap elevation, tissue pliability, and healing after a facelift.

RFMN energy settings can be optimized to balance skin tightening with control of fibrosis by titrating energy per needle, pulse duration, and depth to achieve sufficient dermal coagulation for neocollagenesis and elastogenesis while avoiding excessive thermal injury that may promote fibrotic remodeling. Data supports targeting moderate energy settings (eg, energy per needle 20‐60 mJ, pulse durations 100-300 ms) and limiting the number of passes to induce controlled dermal coagulation, maximizing skin tightening while minimizing the risk of fibrosis [11,12]. Adjusting needle depth to target the reticular dermis and using insulated needles can further localize RFMN thermal effects, thus reducing epidermal damage and unwanted fibrosis [13,14]. Sequential or pulsed energy delivery, as well as energy feedback systems, can help regulate tissue response and prevent overtreatment [15,16].

### Effects on Future Elective Facelift Procedures

Surgical facelift procedures rely on the manipulation of the superficial musculoaponeurotic system (SMAS), a fibrofatty connective tissue layer continuous with the superficial cervical fascia, connected to the platysma muscle inferiorly and the galea superiorly [17]. It plays an integral role in the anatomic relation of the superficial dermis to the underlying facial muscles. There are two distinct SMAS compositions given anatomic regions, and the abrupt junction of differing compositions resides at the nasolabial fold region, where medially, there are fewer fat lobules and a more direct connection of the SMAS to the superficial dermis as muscle fibers are seen to extend superficially into the dermis [17]. However, the other regions of the SMAS lateral to the nasolabial fold still carry the same properties of communication of facial muscle to skin by muscle tendon fibers connecting both regions via the SMAS [18]. Beneath this layer, the SMAS has a complex relation with deep ligaments and connections that limits the mobility of superficial structures. These connections are crucial to release to generate the most optimal movement for desired facelift outcomes [17].

Due to the sophisticated relationship of neighboring structures, RFMN, particularly at greater depths, may alter these structural relationships. Traditional surgical facelifts target a single plane of tissue in a primary horizontal plane [17]. On the contrary, RFMN targets a small treatment area in a vertical configuration through multiple planes of tissue, potentially leading to increased tissue adhesions, difficult surgical dissection, impaired flap mobility, and suboptimal facelift outcomes [10].

Although undergoing RFMN treatments prior to a facelift could potentially induce a level of fibrosis that may help delay the timeline when a patient would be a candidate for a facelift, evidence suggests RFMN may be more beneficial postoperatively. Following a facelift, RFMN could enhance skin tightening and improve aesthetic outcomes by stimulating additional collagen production either as an immediate adjunctive therapy or as a method to combat recurrent long-term skin laxity [19]. Careful planning is necessary to determine the appropriate time frame between treatments to avoid excessive fibrosis and impaired wound healing.

### Patient Considerations for RFMN Procedures

When considering therapy using RFMN, specific patient populations should be considered when discussing treatment options, as certain age groups, as well as patients with jowl laxity, have been shown to experience better outcomes with facial surgery [19]. Taking into consideration that each treatment has varying mechanisms and different anatomical targets, a comparative study found that surgical facelifts improved skin laxity by 46% relative to baseline, whereas RFMN alone achieved only a 16% improvement [20]. These findings underscore the importance of setting realistic patient expectations regarding treatment efficacy. Additionally, patient age should be considered, as older individuals (≥55 years) experience more pronounced skin tightening with RFMN compared to younger patients [2]. Younger patients typically have a higher collagen content in their skin compared to older patients, who undergo collagen loss due to age; thus, the relative resulting decrease in skin laxity is much more noticeable in older patients versus younger patients. This knowledge prompts early discussion of RFMN treatment to address its potential effectiveness or lack thereof, especially at a younger age.

In addition to considering a patient’s age, premature neck and jowl laxity are common concerns among patients seeking skin-tightening treatments. However, RFMN does not effectively target subplatysmal fat, necessitating careful patient selection—individuals with significant subplatysmal fat may achieve superior results with surgical interventions such as liposuction [19]. For patients with pronounced skin laxity, RFMN alone may be insufficient and could exacerbate sagging if incidental heat-induced fat loss occurs without concurrent skin excision [19]. Other complications of RFMN reported include hyper- or hypopigmentation of treated skin, thermal burns, blistering, and scarring; these can often be mitigated with proper technique and equipment settings [8].

## Discussion

### Main Findings

The nuances of RFMN in facial rejuvenation necessitate a deeper understanding of its implications for future surgical facelifts. This calls for detailed discussion between the patient and health care provider to improve pretreatment consultation, patient education, and results. Patients should be informed that RFMN may lead to dermal fibrosis, tissue adhesions, subcutaneous adipose denaturation, and altered SMAS composition, which could complicate facelift procedures and their desired outcomes. Additionally, providers should address the limitations of RF to effectively target jowl laxity and set realistic expectations regarding RF results in patients younger than 55 years. Understanding these points would allow for individualized treatment planning, ensuring patients receive the most appropriate interventions based on their anatomical considerations and aesthetic goals. Furthermore, current evidence suggests RFMN may be better positioned as a postoperative adjunct rather than a presurgical intervention, especially in patients known to be surgical candidates in the future.

It is also important to note that RFMN has primarily been used for skin rejuvenation, mild laxity, and conditions such as acne scars, rather than as a substitute for surgical facelift procedures [5]. Additional systematic reviews and clinical trials, such as those by Austin et al [21] and Nguyen et al [11], further support that early RFMN and radio frequency protocols are designed for modest rejuvenation, targeting mild-to-moderate laxity and stimulating collagen production. Among these studies, reported patient satisfaction was highest among those seeking subtle improvements rather than facelift-level results.

### Limitations

This scoping review is limited by the minimal availability of long-term studies specifically examining the effects of RFMN on subsequent surgical facelift procedures. The available literature mainly consists of case reports, small-scale studies, and expert opinion, which restricts the generalizability of conclusions. In addition, the large amount of variation between RFMN device settings, treatment protocols, and patient demographics across studies further limits the ability to standardize findings or establish definitive treatment guidelines.

### Conclusion

Cosmetic surgical providers trained in RFMN and/or facelift procedures should give careful consideration to this new technology when discussing different options for facial rejuvenation. Factors to weigh in these considerations should include, but are not limited to, age-related expectations, area of treatment, and the potential impact of subsequent facelifts. Rather than viewing RFMN and surgical facelifts as isolated interventions, providers should consider how early noninvasive treatments may influence future surgical options. This has important implications for clinical decision-making, patient education, and informed consent.

Ultimately, optimizing aesthetic outcomes will require a more integrated strategy that aligns patient goals with both immediate and long-term treatment trajectories. Future studies are needed to evaluate the cumulative impact of RFMN on facial anatomy and to guide safe, evidence-based treatment planning for patients considering both noninvasive and surgical facial rejuvenation options. Additional research should also focus on establishing guidelines for the optimal timing of RFMN relative to surgical facelifts and identifying strategies to minimize adverse effects of RFMN. As RFMN technology evolves, ongoing studies will be critical in refining its role in facial rejuvenation and improving patient outcomes.

## Supplementary material

10.2196/78385 Checklist 1 PRISMA-ScR checklist.

## Abbreviations

EGF: epidermal growth factor
FGF: fibroblast growth factor
PDGF: platelet-derived growth factor
PRISMA-ScR: Preferred Reporting Items for Systematic Reviews and Meta-Analyses extension for Scoping Reviews
RF: radio frequency
RFMN: radio frequency microneedling
SMAS: superficial musculoaponeurotic system
TGF-β1: transforming growth factor β-1
TGF-β2: transforming growth factor β-2
TGF-β3: transforming growth factor β-3
